# 

**Published:** 1880-01

**Authors:** 


### INDEX

TO THE

### INDEPENDENT PRACTITIONER.

### VOLUME I.

### *Original Communications.*

PAGE.

Asphyxia, A "Speedy Method" in By H. L. Byrd, M. D.......... 11
Audiphone, The. By J. Allen Osmun, M. D...... ...... ...... ..........135

Blood-Letting in Disease. By Harvey L. Byrd, M. D.............476

Cancer Cures. By Horace R. Mooney, M. D...... ...... ............523

Deafness the Result of Sympathy between the Ear, Teeth and Mouth. By Laurence Turnbull, M. D...... ........................133
Dental Education. By George H. Perine, M. D... ...................425
Dental Patients, Our Young. By Charles E. Francis, D. D. S... 24
Dentistry, Ideal. By C. T. Stockwell.......................................191
Destistry, Operative, vs. Mechanical. By J. Allen Osmun.........348
Diagnosis. By B. F. Coy, D. D. S..............................................240
Diphtheria, Constitutional Origin of. By R. M. Griswold, M. D.270
Diphtheria, Some Practical Suggestions in the Treatment of. By R. J. Nunn, M. D...................................................................415
Duty, Our. By J. Allen Osmun........ ..................................427

"Every Tooth Containing a Metallic Filling is a Galvanic Battery in Active Operation," etc. By Henry S. Chase, M.D., D.D.S. 77
Electro-Therapeutics. By Edward C. Mann, M. D............232, 343
Ethnological Phenomenon, An. By H. L. Byrd, M. D.............375
Ethnological Phenomenon, The. By J. C. Beckham, M. D......438

Flagellation of the Child's Back Previous to its Complete Delivery, as a *Preventive* of Uterine Hemorrhage.

Flagellation of the Abdomen of the Woman after the Delivery of the Placenta, as a *Substitute* for the introduction of the Hand into the Uterine Cavity of the Uterus. By Isaac E. Taylor, M. D. .......... 53

Flagellation of the Abdomen and of the Back of the Infant, in Relation to Post-Partum Hemorrhage, Observations on Dr. Taylor's Paper on. By Rufus W. Griswold, M. D. .......... 185

Fistula. By G. Halsted Boyland .......... 289

Fracture of the Jaw, with Critical Remarks, Treatment of. By Thomas Brian Gunning, D. D. S. ... 171, 296, 367, 430, 468, 526, 564.

Galvano-Cautery, Clinical Notes on the Use of the. By Wm. A. Byrd, M. D. .......... 14

Gonorrhœal Rheumatism, Some Suggestions in Regard to the Treatment of. By Wm. A. Byrd, M. D. .......... 422

Hip-Joint Operation. By G. Glanville Rusk, M. D. .......... 511

Hydrobromic Ether in Labor, The use of. By Laurence Turnbull, M. D. .......... 136

Hydrobromic Ether as an Anæsthetic for Surgical Operations, On the Use of. By Laurence Turnbull, M. D. .......... 74

Hygiene, The, De Lesseps Canal in its Relation to. By G. Halsted Boyland, M. D. .......... 317

Jaw Fractures, Gunning on. By Prof. Jas. E. Garretson, M. D. .......... 562

Medical Schools of Baltimore, The. By Eugene F. Cordell, M. D. .......... 519

Medication, Specific. By Dudley M. Culver, M. D. .......... 242

Meeting of the Georgia Medical Society of Savannah .......... 196

Mysophobia. By William A. Hammond, M. D. .......... 115

Orchitis, Treatment of, with note as to its Varieties, Pathology, Prognosis, Treatment, &c. By G. Halsted Boyland, M. D. ... 463

Poisoned by Vegetables from Poisoned Soils. By W. R. Monroe, M. D. .......... 575

Purpura Hemorrhagica, Case of, Resulting in Dropsy. By Dudley M. Culver, M. D. .......... 426

Rheumatism Acute, in a Child. By Barton Dozier, M. D.........194
Riggs Disease, More About. By George A. Mills....................377
Roots of Teeth, Filling. By Samuel D. Rambo, D. D. S..........524

Scarlatina, Cold Pack in. By D. M. Culver, M. D....................242
Sexual Exhaustion (Sexual Neurasthenia, The Symptoms of. By George M. Beard, M. D..........................................221, 271
Surgery, Compression and Immobilization, Principal Factors in. By G. Halsted Boyland, A. M., M. D............................. 1
Surgeons of Baltimore and their Achievements, The. By B. Bernard Browne, M. D..................................................514, 559

Tetanus — Epidemic or Constitutional among Negroes? By R. H. Goldsmith, M. D..................................................... 22
Teeth, Pregnancy and Disease. By Basil M. Wilkerson, D. D. S. M. D................................................................ 26
Tooth, No Battery in a. By Charles A. Mayr, A. M................284

Water, Its Relation to Health and Disease. By Wooten M. Wilkerson, A. B., M. D.............................................. 4
Water Conductors and the Artesian Well at Charleston, S. C. By Middleton Michel, M. D.......................................126
Wire Gause Supporter, For the Treatment of Fractures of the Lower Extremities. By Harvey L. Byrd, M. D............... 71

Yellow Fever Prevention, Practical Hints Relating to. By R. B. S. Hargis, M. D..................................................334

### *Editorial Department.*

Addendum and Errata..................................................110
Advertiser's Communications, etc....................................... 47
Advertising Physicians..................................................308
American Gynæcological Association................................485
Asphyxia in the Drowned, etc.........................................207
Associations, Fees, Contracts, etc...................................... 93

Baltimore, Geographical Position of..................................391
" Letter, A............................................................444
" Sewerage of, and the National Board of Health.........579

Bandage, Martin's Elastic........................353
Bound Volume for 1880........................539
Buchanan Redivivus........................483
Burns and Scalds........................389

Cancer, Chian Turpentine in........................388
"Cat in a Meal Tub"........................262
Centennial, Our Sesqui........................487, 535
Chloroform, Deaths from........................96
Contributors, A Full Index List of........................582
Correspondents, To........................582
Cough, Oxalate of Cerium as a Remedy in........................390
Craniotomy........................538
Criticism........................359

Dental Friends, To Our........................154
Dental Societies, Meetings of........................264
Dental Society, Tenth Annual Session of the New Jersey State...311
Dental Association, National........................360
Dentists, Hall for a Mass Convention of the........................361
Dental Association, The American........................362
Diploma Vender gone, The Chief........................448
Druggists and Physicians........................255

Epizootic, The Equine........................537
Ethyl, Bromide of........................357

Female Attendents in Stores, Seats for........................448
Females, Occupations suitable for........................481
Former Pupils and Subscriptions to the Journal........................388
Friends and Correspondents, To Our........................209

Hospitals, Their Institution, Management, etc........................148
" Their Location, etc........................205
" In Baltimore........................257
" The City........................440
Hygiene........................308
Hypophosphites, Syrup of........................581

Independent Journalism........................92, 259
Indigent Sick, Care and Attention to the........................304

Johnston Bros. Excursion, The....492
Journal, An Original....357

Lactopeptine....442
Life Insurance Associations.... 46

Marine Hospital for Baltimore....306
Medical Fees, Free Dispensaries, etc....150
Medical and Dental Societies, Papers Read Before....151
" Universities and Colleges, Some of Our....208, 305
" Society of Georgia, Proceedings of the....209
" and Dental Societies, Meetings of....215
" and Chirurgical Faculty of Maryland....263
" Centre, Prospective Advantages of Baltimore as a....407
" Education, Reform on....352, 384
" Association, The American....355
Medico-Literary Journal, The....355
Medical Rats....356
Medicines, Incompatible....356
Medical Service, The Night....391
" Fees and Under Bidding....443
" Schools, Opening of the....537
" Society of Kentucky, Minutes of the Twenty-Fourth and Twenty-Fifth Annual, Meetings of the State....538
" Hall for Baltimore.... 91
Metric System, The....353
Mortuary Statistics....260
" Reports, Our....538
" and Thermometrical Reports....448

Nourishment, Water and Air as....386

Our Second Volume....536

Physicians' Reports of Births, &c....387
" and Dentists, Mutual Life Associations for....485
Practitioner, The.... 95
Prospectus.... 45
Puerperal Eclampsia, Tincture of Aconite Root and Veratrum Veride in....537

Religion, Infidelity and Contempt of........................309
Respiration, The Analgesic Effects of Rapid and Forcible.........390
Ruth vs. Reuling........................358

Specialties and Specialists........................204, 263
Sphygmoscope, A New........................484

Teaching and Licensing........................578
To Former Students........................307
Translations........................48-95
Trommer's Extract of Malt........................442
Turpentine, Chian........................483

Underwriter........................96
University of Maryland, etc........................152
" of Pennsylvania........................157

Veratrum Veride, a Sure Antidote to Opium Poisoning..........388
Volume First, Close of........................580
Volume, Our Second........................536

Water, Pure Drinking........................306
Who are They?........................358
Who was It?........................483

Yellow Fever........................484

### *Excerpta.*

Addendum and Errata........................110
Amalgams, Mercurial........................552
Aphorisms, Professional........................596
Anæmic Menorrhagia........................160
Attenuations, Collecting Bills, Pruritus Ani........................161
Anti Neuralgic: Menthol, A New........................587
Army and Navy, Dentists in the........................494

Bacteria, Influence of Electricity on........................544
Beef Tea and Its Value........................554
Blood Stains........................592
Brain Growth, Domestication in........................170
Bromides, Therapeutic Uses of the........................505

Cæsarean Section on a Dwarf......................................550
Child-birth After Ovariotomy......................................103
Chinese Women, Compression of the Feet of......................600
Chloroform, Atropia against Cardio-Inhibitory Effects of............602
Consciousness, The Evolution of......................................455

Dead Bodies, New Method of Preserving...............................102
Dentistry, A Letter of Importance to Every One Interested in...113
" Of Individual Importance to Every One Interested in, 166, 167, 169
Disinfection......................................................405
De Lessep's Canal in its Relation to Hygiene......................496
Dentists in America..............................................502
Diphtheria Discussion at the Medical Society, The..................598
Diphtheria, Value of Benzoate of Soda in..........................105
Diarrhœa a Zymotic Disease........................................104
Doctors vs. Apothecary, A Crusade Against Quackery...........158

Enema, Domestic..................................................410
Ether in Sciatica, Subcutaneous Injection of......................588

Fecundity, Extraordinary...........................................413
Female Physicians In The Olden Time...............................459
Fissure, Treatment of Anal........................................461
France and Germany, Population Statistics of.......................407

George Washington, The Death of...................................459

Headache, Remedies for............................................589
Health, The National Board of...............................101, 401
Hepatic Calculi, Treatment of.....................................104
Hydrophobia, Transmission of, From Men to Rabbits...............100

Iodine a Substitute for Quinia ....................................395
Iodide of Starch in Poisoning.....................................165
Iodoform Suppositories for the Urethra, and Canal of the Cervix Uteri.............................................................501
Ingrowing Nails, Painless Operation for............................554

Jaborandi in Dropsy, Quebracho in Dyspepsia........................159

Labor, Should We Bandage After, and Why....................555
Labor, Anæsthetics in..................................................604

Maize, Stigmata of......................................................500
Measles not a Trivial Disease......................................412
Medical Charity, and What Comes of it ........................101
Medical Services, Night...............................................405
Medical Registration Law, The.....................................503
Medical Association, American, Transactions of..............212
Metric System Has Been Getting On, How The..............592
Melanosis, a Case of in Philadelphia............................410
Microcopists, Physicians as.........................................106
Menses, Restoration of................................................106

Near Sight in the Young, Prevention of..........................545
Nervous Exhaustion, Treatment of Chronic.....................159

Opium Habit, Coca as an Antidote for the.......................402

Patients, Gratitude of..................................................598
Paraphimosis, Simple Method of Reducing....................600
Perineum, Prevention of Lacerated...............................460
Phthisis, Chloride of Calcium in the Treatment of............411
Physicians, Duty of.....................................................507
Physicians, Mortality of...............................................605
Phthisis, Creosote in...................................................591
Physician in the School Room, The................................601
Pregnancy, Positive Sign of During the First Three Months......553
Poisoning, Iodide of Starch in......................................165
Perineum, The obstetric Treatment of............................400
Profession, How to Succeed in Our................................145
Prince Imperial, Identification of the...........................108
Postal Law................................................................315
Potash, Salicylate of....................................................315
Potassium, Bromide of.................................................461
Pleuritis Effusion.—Treatment by Hypodermic Injections of Pilocarpin..........................................................599
Pulmonary Phthisis Treatment of, by Benzoate of Sodium.........602

Rapid Breathing as a Pain Obtunder in Minor Surgery, Obstetrics, The General Practice of Medicine and of Dentistry............398
Respiration, Artificial..........................................................408

Scarlet Fever, Action of Benzoate of Soda in..........................498
Selections...........................................................................509
Shampooing Advisable? Is.....................................................605
Ship Railway, Capt. Eads'.....................................................543
Smoke, Learning to..............................................................543
Substitute for Quinia, Iodine a...............................................395
Sweating Hands or Feet........................................................162
Surgery, Antiseptic...............................................................597

Tanner Fast, The..................................................................460
Teeth, Thomas, on Hearing Through the...................................495
Teeth, To Bleach Discolored..................................................551
Teeth, Bleaching..................................................................409
Therapeutics, German..........................................................105
Tit for Tat...........................................................................216
Tonga,...............................................................................404
Tracheotomy Superseded......................................................506
Typhoid Fever, Iodine in.......................................................588
Typhoid Fever, Antiseptic treatment of...................................595
United States Postal Law.......................................................315

Urine in Children, Incontinence of..........................................107

Water Trap, The Efficiency of the...........................................409

Yellow Fever, Doctors In Memphis During...............................159

### *Bibliographical Department.*

Artificial Anesthesia, The Advantages and Accidents of...........154
Aspiration of the Knee Joint...................................................266

Exhaustion, A (Neurasthenia,) Its Symptoms, Nature, Sequence and Treatment.........................................................................264

Headaches, Their Nature and Treatment..................................210

Hygiene and Sanative Measure for Chronic Catarrhal Inflammation of the Nose, Throat and Ears....................................541

Investigation and Experience of M. Shawtinbach at Saar Soong, Sumatra....................................................................312

Library of the Surgeon General's Office U. S. Army, Index Catalogue of the..............................................................487

Mechanical Dentistry, A Practical Treatise on..........................585
Mammory Gland, Practical Treatise on the, Embracing Their History, Diagnosis and Treatment...................................450

Periodicals, Pamphlets, etc................................................585
Pamphlets, Periodicals, etc...........................................541, 542
Periodicals, Pamphlets, etc...........................................212, 216
Prolonging Life, The Art of...............................................582
Physicians' Visiting List, The.............................................542
Physiology, A New School..................................................450
Periodicals, Pamphlets, etc...............................................96, 100
Periodicals, Pamphlets, etc...........................................154, 157
Periodicals, Pamphlets, etc...........................................266, 269
Periodicals, Pamphlets, etc...........................................312, 314
Periodicals, Pamphlets, etc.............................364, 365, 393, 394
Preadamites.....................................................................540
Practitioners' Reference Book, The.....................................488

Sea-Sickness, A Practical Treatise on, Its Symptoms, Nature and Treatment..........................................................................315
Skin in Health and Disease, The.........................................489
Surgery in the Pennsylvania Hospital...................................363

Treatise on Oral Deformities as a Branch of Mechanical Surgery, a..........................................................................449

Yellow Fever, A Nautical Disease........................................210

### *Translations.*

Dental Caries...................................................................244

Progress, Medical.............................................................. 82

Spermatic Hydrocele, (From the German)........................137
Sulphite of Quinia, Physiological action of........................140

### *Correspondence.*

Advertising in Medical College........................576
Announcements and Newspapers, Chisolm-Reuling Embroglio, The........................480

Belladonna Causes Dryness of the Mouth........................439

Pure Bath Rooms and Clean Water Closets........................539

To Clean Teeth, How, ℞ 1568........................330

### *Eclectic Department.*

Dentine, Obtunding Sensitive........................552
Dyspepsia and the Teeth........................253

Extirpation of the Nose and Mouth by the use of the Surgical Engine, By D. H. Goodwillie, M. D., D. D. S........................ 28

Gold Fillings Sixty-four Year Old........................147
Gout, Subacute and Chronic, Treatment of, By Alex. Hadden, M. D........................ 247

Medical Charities, Abuse of, and Unprofessional Conduct of Medical Examiners........................198
Miasm and Fevers........................ 87
Molar, An Intractractable, By P. K. Stoddard, M. D........................ 44
Musical Heart Murmurs, By J. M. Da Costa, M. D........................ 84

Rheumatism, On the Therapeutics of Acute, By Roberts Bartholow, M. D........................ 37

Tooth, Caries of Pregnancy, By Edward C. Kirk, D. D. S........................200
Teeth, Re-plantation of........................ 89
Toothache, A Pleasant Remedy for, By T. C. Osborn, M. D........................ 49

### *Hymeneal.*

Morris—Greenwood......108 Judkins—Anderson......162 Hopkins—Roberts......217 Bergen—Slivus......269 Blake—Marriott, ......314 Dodge—Lodge......314 Claude—Wilkerson......365 Galt—Randolph......394 Barden—Lednum......455 Early—Veasy ......492 Hunter—Henderson......493 Turnes—Daughty......542 Wood—Davis......585

### *Natalitial.*

Anderson, B. P., Jr......108 Hill, Charles G.......162 Byrd, Harvey L......217 Wayman; E. F......269 Culver, Dudley M......493 Davis, J. E......586 Wilkerson, Charles......586

### *Necrological.*

Freeman, J. Bunstead......109 White, Samuel S......109 Toland, H. H......162 Lawrence, Mordecai.....162 Bullitt, Henry M......162 Weikel, Chas. H......162 Cooper, Tunis......217 Goersen, Geo. F......217 Hailes, A. B......217 Ward. J. A......517 Thomas, D. G......217 Darrell, J. H......217 Choppin, Samuel......269 Sayre......269 Keener, Wm. H......314 Seymour, E. W......365 Armstrong, H...365 Morris, Richard Lewis...394. Preston, B...395 Taylor, Alfred Swaine......455 Hauser, William......493 Rawland, James W......242 Seguin, Edward......686 Arthur, Robert......586

### *Mortuary and Meteorological.*

Pages......50, 52, 111, 113, 163, 165, 218, 219, 270, 316, 366, 460, 110, 558, 606

Special Notice......220

### PROSPECTUS
OF
### THE PRACTITIONER.
AN
### INDEPENDENT MONTHLY JOURNAL,
*DEVOTED TO MEDICAL, SURGICAL, OBSTETRICAL AND DENTAL SCIENCE.*

The fixed purpose of the projectors of this journal to adhere with unvarying fidelity to independence in all matters appertaining to its conduct and management, renders it unnecessary to issue a lengthy prospectus of its objects and aims. With a few brief statements, therefore, it will be launched forth upon the sea of journalism to take its chances for public favor among the many craft now sailing more or less propitiously thereon. While it respectfully invites subscriptions from every quarter, it naturally looks for primary assistance from old friends and acquaintances. It will know no sectionalism, and, therefore, confidently expects to merit the friendship and good-will of those who *really* desire to sustain and keep from degradation to the level of the merest trade, the grandest and noblest calling known to humanity. From all such, subscriptions, contributions on subjects of interest to the profession, and advertisements are respectfully solicited. The experience of the senior editor, in the past, will be brought into activity in catering to the taste of its readers, and nothing will ever mar its pages not consistent with the standard that has been erected for its conduct and management. Being entirely unconnected with colleges and cliques, it will be bold, fearless and honest in speaking and upholding the right in *all* matters coming within its purview, and will scrupulously render even-handed justice to every one. All subjects of interest to Physicians, Obstetricians, Surgeons and Dentists will claim the attention of its editors and find space in its pages. To this end translations from the French and German periodicals will be introduced from time to time, and the English and American journals made tributary to its columns. All necessary arrangements have been made to *secure permanency* to the enterprise, and to keep it the independent echo and exponent of all that is new and good in the field of its labors. Exchanges of Medical, Dental and other scientific journals are respectfully requested from all parts of the world. Exchanges and books, except Dental, for notice or review, should be addressed to the senior editor, and Dental exchanges, subscriptions, advertisements, and *all* matters relating to the business management of the journal, should be sent invariably to the junior editor and proprietor.

HARVEY L. BYRD, A. M., M. D.,
*No. 147 Edmondson Avenue.*
BASIL M. WILKERSON, D. D. S., M. D.,
*No. 68 North Charles Street.*

